# Prevalence of pain and its associated factors among the oldest-olds in different care settings – results of the AgeQualiDe study

**DOI:** 10.1186/s12875-018-0768-8

**Published:** 2018-06-09

**Authors:** Tina Mallon, Annette Ernst, Christian Brettschneider, Hans-Helmut König, Tobias Luck, Susanne Röhr, Siegfried Weyerer, Jochen Werle, Edelgard Mösch, Dagmar Weeg, Angela Fuchs, Michael Pentzek, Luca Kleineidam, Kathrin Heser, Steffi Riedel-Heller, Wolfgang Maier, Birgitt Wiese, Martin Scherer, for the AgeCoDe & AgeQualiDe study group, Wolfgang Maier, Martin Scherer, Hendrik van den Bussche, Heinz-Harald Abholz, Christian Brettschneider, Cadja Bachmann, Horst Bickel, Wolfgang Blank, Sandra Eifflaender-Gorfer, Marion Eisele, Annette Ernst, Angela Fuchs, André Hajek, Kathrin Heser, Frank Jessen, Hanna Kaduszkiewicz, Teresa Kaufeler, Mirjam Köhler, Hans-Helmut König, Alexander Koppara, Diana Lubisch, Tobias Luck, Dagmar Lühmann, Melanie Luppa, Tina Mallon, Manfred Mayer, Edelgard Mösch, Michael Pentzek, Jana Prokein, Steffi G. Riedel-Heller, Susanne Röhr, Anna Schumacher, Janine Stein, Susanne Steinmann, Franziska Tebarth, Carolin van der Leeden, Michael Wagner, Klaus Weckbecker, Dagmar Weeg, Jochen Werle, Siegfried Weyerer, Birgitt Wiese, Steffen Wolfsgruber, Thomas Zimmermann

**Affiliations:** 1 Department of Primary Medical Care, Center for Psychosocial Medicine, University Medical Center Hamburg-Eppendorf, Hamburg, Germany; 2 Department of Health Economics and Health Services Research, Hamburg Center for Health Economics, University Medical Center Hamburg-Eppendorf, Hamburg, Germany; 3 Institute of Social Medicine, Occupational Health and Public Health (ISAP), University of Leipzig, Leipzig, Germany; 4 Central Institute of Mental Health, Medical Faculty Mannheim, Heidelberg University, Mannheim, Germany; 5 Department of Psychiatry, Klinikum rechts der Isar, Technical University of Munich, Munich, Germany; 6 Institute of General Practice, Medical Faculty, Heinrich-Heine-University Düsseldorf, Düsseldorf, Germany; 7 Department of Psychiatry, University of Bonn, Bonn, Germany; 8 Work Group Medical Statistics and IT-Infrastructure, Institute for General Practice, Hannover Medical School, Hannover, Germany; 9 DZNE, German Center for Neurodegenerative Diseases, Bonn, Germany

**Keywords:** Prevalence of pain, Impairment in daily activities, Care setting, Primary care

## Abstract

**Background:**

The prevalence of pain is very common in the oldest age group. Managing pain successfully is a key topic in primary care, especially within the ageing population. Different care settings might have an impact on the prevalence of pain and everyday life.

**Methods:**

Participants from the German longitudinal cohort study on Needs, Health Service Use, Costs and Health-related Quality of Life in a large Sample of Oldest-old Primary Care Patients (85+) (AgeQualiDe) were asked to rate their severity of pain as well as the impairment with daily activities. Besides gender, age, education, BMI and use of analgesics we focused on the current housing situation and on cognitive state. Associations of the dependent measures were tested using four ordinal logistic regression models. Model 1 and 4 consisted of the overall sample, model 2 and 3 were divided according to no cognitive impairment (NCI) and mild cognitive impairment (MCI).

**Results:**

Results show a decline in pain at very old age but nonetheless a high prevalence among the 85+ year olds. Sixty-three per cent of the participants report mild to severe pain and 69% of the participants mild to extreme impairment due to pain with daily activities. Use of analgesics, depression and living at home with care support are significantly associated with higher and male gender with lower pain ratings.

**Conclusions:**

Sufficient pain management among the oldest age group is inevitable. Outpatient care settings are at risk of overlooking pain. Therefore focus should be set on pain management in these settings.

## Background

Due to physical or mental illness or disability 42% of women and 29.6% of men aged 85–89 years receive care in Germany (care is termed according to the German law SGB XI). This number increases again for the 90+ year-olds (67.9% women, 51.8% men) [1]. These numbers show that with the aging population more attention has to be paid to prevalence and management of pain which goes beyond the treatment with analgesics only and includes alternative options such a relaxation techniques or biofeedback in the oldest age group (85+ years). The presence of daily pain leads to a decline on activities of daily living, especially for cognitive impaired individuals [2–5]. Lower levels of education, female gender and a high Body Mass Index (BMI) seem to entail acute and/or chronic pain [2, 6–8]. Individuals reporting more severe pain were found to score significantly lower in memory tests, for executive function and showed an impaired attentional capacity [2] which has an impact on quality of life and one’s independence. Along with other special needs developed with growing age such as geriatric syndromes, frailty, comorbidities, falls and functional decline [9–12] the use of a care service or moving to a care facility is an inevitable consequence in many cases.

The German health care system supplies care at home either with the help of a relative or through a mobile care service. Otherwise moving to a care home or to a facility with “assisted living” (Betreutes Wohnen) which aims at maintaining one’s independence by providing helpful services e.g. barrier-free apartment, emergency call, meals and a quick access to care support are other options if care is needed [1].

Acute pain is experienced regularly by up to 49–83% of elderly individuals above the age of 60 living in care homes and 40% of elderly individuals living in the community [13, 14]. Each care setting has its own challenges in dealing with pain and pain management such as shortness of time, lack of staff or lack of knowledge [15] but the effects on pain and the apparent housing situation have not been studied in depth yet. However, studies revealed that patients above the age of 85 were found to be less likely to receive adequate analgesic treatment or even none at all [16] and inadequate pain assessment [6, 17, 18]. Very few studies include the group of over 85 year-olds in their research. Therefore we aimed at uncovering underlying differences in pain prevalence and management and hence our research questions focused on the prevalence of pain in the oldest age group with emphasis on the housing situation, on associated factors with pain and the impact of pain on activities of daily living.

## Methods

### Study design and sampling

The data were derived from the German longitudinal cohort study on Needs, Health Service Use, Costs and Health-related Quality of Life in a large Sample of Oldest-old Primary Care Patients (85+) (AgeQualiDe) which is considered a follow-on study (follow-up 7 to 9) on Ageing, Cognition and Dementia in Primary Care Patients (AgeCoDe) (75+). In our analysis, we provide cross-sectional results from the baseline of AgeQualiDe that is the 7th follow-up of AgeCoDe. For the initial study 3327 patients had been recruited from over 138 general practitioners (GP) in six German cities (Bonn, Düsseldorf, Hamburg, Leipzig, Mannheim, Munich) in 2003. All GP patients who participated in the study provided written informed consent prior to their participation. Both studies – AgeCoDe and AgeQualiDe – have been approved by the ethics committees of all participating study centres and comply with the ethical standards of the Declaration of Helsinki. Criteria for inclusion in the original study in 2003 were aged 75 and over, the absence of dementia and a minimum of one contact with the GP per year. Patients had been excluded if one of the following aspects applied: GP consultations only by home visits, lack of the German language, a severe illness with an anticipated fatal outcome within three months, blindness, deafness, inability of consent, residence in a nursing home and not being a patient of the participating GP. The study design of AgeCoDe has been described in detail elsewhere [19]. Within the AgeCoDe study six follow-up waves have been carried out in 1.5 year intervals. With the beginning of follow-up wave 7/ AgeQualiDe baseline 868 individuals are still remaining in the sample. The missing individuals had died before this study wave, refused participation, dropped-out or were otherwise unable to participate. From the baseline sample 757 individuals scored 19 or above in the Mini-Mental-Examination and from them 738 completed the PRS. Mean age of the participants was 88.8 years (SD 2995).

### Measures

#### Pain

The pain assessments were part of a structured clinical interview by trained physicians and psychologists during visits to the participants’ homes. Participants were presented with two questions based on the validated version of the German Brief Pain Inventory [20]. First participants had to rate today’s severity of pain on a one-dimensional numeric pain rating scale (PRS) ranging from 0 to 100 (no pain to worst pain imaginable). Participants were then grouped and assigned to four categories: “no pain” for participants scoring 0 in the PRS, “mild pain” when scoring between 1 and 30, “moderate pain” when scoring 31–60 and “severe pain” when scoring 61 and above in the PRS. Secondly, participants were asked to rate the impairment with daily activities caused by pain in the last 24 h on a five point Likert-scale, ranging from 1 (no impairment) to 5 (extreme impairment). Furthermore, the use of analgesics was assessed by recording the name and number of all prescribed and over-the-counter drugs the participant used in the last 3 months. All drugs belonging to the Anatomical Therapeutic Chemical–Code subgroup N02 were included in the analgesic use evaluation in our analysis.

#### Demography and housing

Items on sex, age, Body Mass Index (BMI), level of education and the current housing situation were assessed. Participants were grouped into two age brackets: up until 89 years old and 90+ years old. For BMI participants were grouped into underweight = < 18.5, normal weight = 18.5–24.9, overweight = 25–29.9 and obesity = BMI of 30 or higher. The level of participant’s education has been categorized into primary, secondary and tertiary according to the CASMIN criteria [21]. The housing situation was assessed during the interview by the question “Do you live alone or together with another person in a common-household?” Participants were grouped into “living at home without care support”, “living at home with care support”, “living in a care-home” and “assisted living”.

#### Cognitive function and clinical variables

Cognitive function was assessed using the Mini-Mental State Examination (MMSE) [22] to detect cognitive impairment. MMSE score below 25 was used as cut-off point for mild cognitive impairment (MCI). Depressive symptoms were measured using the Geriatric Depression Scale [23] consisting of 15 items. A score ranging from 0 to 15 was calculated for each individual (for more details, Weyerer et al. 2008 [19]). Furthermore, the impairments of activities of daily living (IADL) e.g. eating, walking stairs, wash/shower were assessed using the Barthel-Index [24]. A score ranging from 100 (independent from care support) to 0 (dependent on care support) was calculated for each participant.

#### Statistical analysis

Analyses were carried out for participants who scored 19 and above in the Mini-Mental State Examination (MMSE) (*n* = 757) and from whom we assessed the PRS (*n* = 738).

Demographic and clinical characteristics were assessed. Our dependent variables prevalence of pain and impairment with daily living were divided into the groups mentioned above. Associations were tested using Pearson’s □^2^ test for categorical variables, one-way ANOVA for continuous variables and Kruskal-Wallis-test for variables that violated assumptions of normality.

Associations of the dependent measures “prevalence of pain” and “impairment with activities of daily living” were then tested with ordinal logistic regression models adjusting for sex, age, education, housing situation, analgesics, BMI, IADL and Depression. Three models were created for prevalence of pain (1–3) and one model for impairment in daily living (4). All models include all factors and categories apart from MMSE. In model 1 MMSE is included for the overall sample. In model 2 we included participants with MMSE above 25 (non-cognitively impaired group – NCI) and in model 3 for participants with MMSE up to 25 (mild cognitively impaired group – MCI). Model 4 has been calculated for the overall sample for impairment in daily living. It was also repeated for the two MMSE groups but no significant differences appeared. Additionally, interaction effects have been calculated for age and living in a care home but showed no statistical significance. Data for MMSE and interaction effects is given upon request.

SAS 9.3 software was used for logistic regression analysis and SPSS Statistics 23 for the remaining statistical analysis. Statistical significance level was set to alpha 0.05.

## Results

### Demography and prevalence of pain

General and demographic characteristics of our sample are displayed in Table 1. Results are shown for the total sample and stratified by the four categories of pain. Female gender was predominant in the sample (67.5%). Sixty-three per cent of the participants reported mild to severe pain of which 57.5% are male and 65.7% female. Thirty-seven per cent of the participants reported no pain (M: 26.15, SD: 26.68). Assessing the housing situation participants using assisted living show the lowest prevalence of pain (54.2%) followed by care home residents (60%) and participants living at home without care support (61.7%). The highest prevalence of pain was reported by participants living at home with care support (75.2%). Furthermore the latter group reported the highest average pain scores. In this group 39% of the sample reported moderate and 19% severe pain. In contrast, participants living at home without care support and afflicted with pain scored highest in the mild pain category (29.4%).

**Table 1:** Sample of Descriptives and Demography

	All N (%)	No Pain N (%)	Mild Pain N (%)	Moderate Pain N(%)	Severe Pain N(%)	Pearson □^2^
Participants (N)	738	273 (37.0)	195 (26.4)	191 (25.9)	79 (10.7)	
Gender						□^2^ [3]= 19.47 *p* = <.001
Male	240 (32.5)	102 (37.4)	77 (39.5)	42 (22.0)	19 (24.1)	
Female	498 (67.5)	171 (62.6)	118 (60.5)	149 (78.0)	60 (75.9)	
Age (y), M (SD)	88.8 (3.0)					□^2^ [3] = 9.18 *p* = .027
≤ 89	476 (64.5)	163 (59.7)	134 (68.7)	119 (62.3)	60 (75.9)	
≥ 90	262 (35.5)	110 (40.3)	61 (31.3)	72 (37.7)	19 (24.1)	
Education						□^2^ [6] =27.17 *p* = <.001
Primary	408 (55.3)	151 (55.3)	90 (46.2)	117 (61.3)	50 (63.3)	
Secondary	226 (30.6)	91 (3.3)	59 (30.2)	59 (30.9)	17 (21.5)	
Tertiary	104 (14.1)	31 (11.4)	46 (23.6)	15 (7.8)	12 (15.2)	
Housing						□^2^ [9] = 1.89 *p* = <.001
At home^a^	520 (70.5)	199 (72.9)	153 (78.5)	118 (61.8)	50 (63.3)	
At home^b^	105 (14.2)	26 (9.5)	18 (9.2)	41 (21.5)	20 (25.3)	
Assisted living	65 (8.8)	22 (8.1)	11 (5.6)	10 (5.2)	5 (6.3)	
Care home	48 (6.5)	26 (9.5)	13 (6.7)	22 (11.5)	4 (5.1)	
Analgesics*						□^2^ [3] = 5.01 *p* = <.001
Yes	181 (24.5)	33 (12.1)	42 (21.5)	73 (38.2)	33 (41.8)	
No	546 (74.0)	234 (89.0)	151 (77.4)	115 (60.2)	46 (58.2)	
BMI, M (SD)*	25.2 (3.8)					□^2^ [9] = 8.96 *p* = .441
Normal weight	340 (49.1)	129 (47.3)	95 (48.7)	83 (43.5)	33 (41.7)	
Underweight	17 (2.5)	9 (3.3)	3 (1.5)	2 (1.0)	3 (3.8)	
Overweight	265 (38.3)	88 (32.2)	69 (35.4)	79 (41.4)	29 (36.7)	
Obese	70 (10.1)	23 (8.4)	18 (9.2)	18 (9.4)	11 (13.9)	
MMSE						□^2^ [3] = 8.30 *p* = .040
< 25	79 (10.7)	38 (13.9)	16 (8.2)	22 (11.5)	3 (3.8)	
≥ 25	659 (89.3)	235 (86.1)	179 (91.8)	169 (88.5)	76 (96.2)	
Impairment in daily activities*						□^2^ [15] = 521.90 *p* = <.001
None	229 (31.0)	181 (66.3)	42 (21.5)	6 (3.1)	0 (0.0)	
Mild	215 (29.1)	65 (23.8)	91 (46.6)	47 (24.6)	12 (15.2)	
Medium	158 (21.4)	24 (8.8)	50 (25.6)	73 (38.2)	11 (13.9)	
Moderate	113 (15.3)	3 (1.1)	10 (5.1)	62 (32.5)	38 (48.1)	
Extreme	21 (2.8)	0 (0.0)	1 (0.5)	3 (1.6)	17 (21.5)	

Notes:

*M* mean

*SD* standard deviation

Data are presented as number (%)

Alpha was set to 0.05

*Due to missing values not all sums add up to 100%.

^a^Participants without care support.

^b^Participants with care support

Of those participants experiencing pain 67.8% did not take any analgesics at the time. Focusing on the cognitive status 51.9% of participants with MCI and 64.3% of participants with NCI reported pain. This difference reached statistical significance (□^2^ [3] = 4.45, *p* = .035). In total 229 (31%) participants reported no impairment and 509 (69%) participants mild to extreme impairment due to pain with daily activities.

The bivariate analysis were carried out for the measures age, sex, cognitive impairment (MMSE), analgesics, housing situation, Barthel-Index, BMI and education (see Table 1). Significant differences were appeared between the two age groups (□^2^ [3] = 9.18, *p* = .027), for men and women (□^2^ [3] = 19.47, *p* = <.001), for the education groups (□^2^ [6] = 27.17, *p* = <.001), between use and no use of analgesics (□^2^ [3] = 55.01, *p* = <.001) and for the four different housing situations (□^2^ [9] = 31.89, *p* = <.001). No significant result was found for the BMI groups nor for Barthel-Index scores. These variables were then put into the multivariate analysis (model 1 – model 4). Results are presented in Table 2.

**Table 2:** Associations of prevalence of pain for all participants, for participants with MMSE > 25, for participants ≤25 and impairment in daily living for all participants

Variable	Model 1			Model 2			Model 3			Model 4		
	OR	95% CI	*p*	OR	95% CI	*p*	OR	95% CI	*p*	OR	95% CI	*p*
Gender												
Female	-	-	-	-	-	-	-	-	-	-	-	-
Male	0.61	0.45–0.84	<.01	0.61	0.44–0.84	<.01	0.21	0.05–0.93	0.04	0.59	0.43–0.81	<.01
Age												
<=89	-	-	-	-	-	-	-	-	-	-	-	-
> = 90	0.65	0.48–0.88	<.01	0.61	0.44–0.84	<.01	2.17	0.50–9.36	0.30	0.88	0.65–1.19	0.41
Education												
Primary	-	-	-	-	-	-	-	-	-	-	-	-
Secondary	0.75	0.54–1.02	0.07	0.73	0.52–1.01	0.06	4.83	0.71–32.94	0.11	0.76	0.56–1.05	0.09
Tertiary	1.12	0.73–1.71	0.60	1.18	0.76–1.83	0.47	1.31	0.17–10.40	0.79	0.97	0.63–1.48	0.88
Housing												
Living at home^a^	-	-	-	-	-	-	-	-	-	-	-	-
Living at home^b^	1.60	1.01–2.53	0.04	1.70	1.04–2.78	0.04	1.44	0.30–6.90	0.65	1.72	1.08–2.74	0.02
Assisted living	0.71	0.38–1.31	0.27	0.90	0.47–1.71	0.75	<.01	<.001	0.96	0.61	0.33–1.13	0.12
Care home	0.68	0.37–1.24	0.21	0.78	0.39–1.57	0.48	0.08	0.01–0.48	<.01	0.51	0.28–0.93	0.03
Use of Analgesics^c^	2.17	1.48–3.16	<.01	2.13	1.42–3.18	<.01	2.62	0.49–13.98	2.62	2.75	1.88–4.03	<.01
BMI												
Adiposity	1.14	0.71–1.84	0.60	1.17	0.71–1.92	0.53	0.24	0.02–2.81	0.25	1.48	0.92–2.39	0.11
Overweight	1.21	0.89–1.65	0.22	1.19	0.86–1.63	0.30	3.14	0.71–13.99	0.13	1.37	1.01–1.86	0.05
Normal weight	-	-	-	-	-	-	-	-	-	-	-	-
Underweight	0.74	0.29–1.91	0.53	0.55	0.20–1.53	0.25	2.88	0.07–114.06	0.57	1.36	0.05–3.48	0.52
Barthel Index^d^	0.99	0.98–1.01	0.19	0.99	0.98–1.01	0.30	1.01	0.96–1.07	0.64	0.99	0.98–1.01	0.27
Depressive symptoms^e^	1.15	1.08–1.22	<.01	1.14	1.07–1.22	<.01	1.42	1.01–1.99	0.04	1.14	1.07–1.21	<.01
MMSE^f^	0.60	0.35–1.03	0.06	-	-	-	-	-	-	0.69	0.41–1.17	0.17

Notes: *OR* Odds Ratio, Alpha was set to 0.05

^a^Participants without care support

^b^Participants with care support

^c^use of analgesics: dichotomized

^d^Barthel Index: score 0–100

^e^GDS score 0–15

^f^MMSE: score 0–30, cut-off ≤25 for mild cognitive impairment, > 25 no cognitive impairment

### Model 1 – Overall pain ratings

In model 1 we tested the association of demographic and clinical characteristics on the overall sample. Male sex was statistically significantly associated with lower PRS (OR 0.61, 95% CI 0.45–0.84) while contrary associations were found for age. Individuals between 90 to 94 years of age scored significantly lower in the PRS (OR 0.65, 95% CI 0.48–0.88) than the younger age bracket. Considering the housing situation higher PRS was significantly associated with participants living at home with care support. There is a 1.6 higher possibility of experiencing pain for this group than living at home without care support (OR 1.60, 95% CI 1.01–2.53). There was also a significant association for analgesics. Participants receiving analgesics experience twice as much pain (OR 2.17, 95% CI 1.48–3.16). Furthermore higher PRS was significantly associated with depression (OR 1.15, 95% CI 1.08–1.22). No significant evidence showed for differences in education, BMI or Barthel-Index.

### Model 2 and 3 - MMSE status

In model two and three we divided the sample in NCI and MCI participants and found significant associations for both groups: Again male sex was significantly associated with lower pain ratings for both groups (NCI: OR 0.61, 95% CI 0.44–0.85 and MCI: OR 0.21, 95% CI 0.05–0.93). Significant associations for age appeared according to model 1 for NCI aged 90 to 94 years old. The older age group scored significantly lower in the PRS (OR 0.61, 95% CI 0.44–0.84). No association with age was found for the group the MCI-participants. Concerning the housing situation living at home with care support was significantly associated with higher ratings on the PRS for NCI (OR 1.70, 95% CI 1.04–2.78) and significantly associated with lower ratings on the PRS for MCI-participants living in a care-home (OR 0.08, 95% CI 0.01–0.48). Also for both groups higher PRS was significantly associated with depression (NCI: OR 1.14, 95% CI 1.07–1.22 and MCI: OR 1.42, 95% CI 1.01–1.99). Significant associations for analgesics were found for NCI. Those taking analgesics reported twice as much pain (OR 2.12, 95% CI 1.42–3.18). No significant figures appeared for education, BMI or Barthel-Index.

### Model 4 - impairment in daily activities

In model 4 we analyzed the impairment in daily activities for the overall sample. Again we found a significant association for lower impairment in daily activities with male sex (OR 0.59, 95% CI 0.43–0.80), analgesics (OR 2.75, 95% CI 1.88–4.03) and depression (OR 1.14, 95% CI 1.07–1.21). The housing situation showed significant less impairment in daily activities for participants living in a care home (OR 0.51, 95% CI 0.28–0.93) and more impairment for participants living at home with care support (OR 1.72, 95% CI 1.08–2.74). For BMI stronger impairment in daily activities was significantly associated with overweight participants (OR 1.37, 95% CI 1.01–1.86). Education and Barthel-Index did not reach statistical significance.

## Discussion

This was one of a few existing studies focusing on the prevalence of pain and associated factors such as age, gender, use of analgesics, cognitive state, medication and depression among the population of 85+ year olds.

### Age

The study demonstrated a high prevalence of pain (63%) among the age group of 85+ and strong experience impairments with everyday life due to pain (69%). Additionally, our findings suggest that older adults report less pain. These findings are supported by Zyczkowska (2007) [25] who reported decreasing pain scores with growing age for both men and women. The researchers name a changing, more accepting attitude towards pain with increasing age, a “survival bias” meaning only the healthiest managed to reach very old age as possible grounds for these results. In depth research could provide more distinct answers into what leads to these changes in old age and may help overcome fears of ageing.

### Gender

Regardless of the cognitive state, men seem to experience less pain and impairment in everyday life due to pain. According to these findings, female gender could increase the risk of experiencing pain. Similar finding have been reported in literature across all age groups [6, 25, 26]. Reasons can be differences in individual pain perception, personal coping strategies or social settings [27, 28]. Gaining a better understanding of the different kinds of pain may also play an important role and may allow deeper insight of coping strategies and physiological factors in men and women [29, 30].

### Housing situation

Regarding the housing situation, our data show differences for the housing arrangements chosen in late life. In our sample, 47.8% of women and 34.5% of men aged 90+ years are living in care homes where activities of daily living are reduced and support through care staff is given in many areas [1] which may reduce possible pain hazards. Furthermore, it can be speculated that the availability and home visits of general practitioners are at a higher frequency than in any other housing setting which could have positive effects on pain management. It may also explain why on the other hand participants living at home receiving care experience significantly more pain and more impairment in activities of daily living. Health care services providing care in the community are generally under immense time pressure and restriction of duties applied by the indication of care level or previous health care assessments e.g. supplying medication, changing bandaging, wash the person. It is also unclear whether pain assessment is part of health care services in the community by default nor if caring relatives focus on the issue. Hence the differences in pain prevalence point to a gap in pain management in outpatient care settings.

### Analgesics and cognitive state

Besides reducing pain hazards the use of analgesics plays an important role in pain management. Our results show individuals taking analgesics experience more pain and more impairment in everyday life. This connection may be even stronger due to strong side effects caused by analgesics which further reduce quality of life. Regarding the use of analgesics we found that a large group of participants with pain do not receive analgesics at all which could be explained by missing access to direct medical care, embellishment of pain, refusal to take analgesics, acceptance of pain as one part of growing older or changes in cognitive state. Bauer et al. [31] divided their sample similarly into cognitive unimpaired (CUS) and cognitively impaired participants (CI) with the subgroups of verbal ability (CI-V) and inability (CI-NV) to communicate pain. Though cut-off scores for cognitive impairment varied, the findings point into a similar direction. CUS participants were more likely to receive analgesics while CI participant’s risk of not receiving analgesics despite pain indication was significantly increased by up to 2.6 (CI-V) and 3.4 (CI-NV) times. In our sample NCI patients reported twice as much pain despite receiving analgesics. One would have assumed that individuals who are verbally able to express their pain and receive the accurate medication should no longer suffer from pain. This instance may have occurred due to chronic or worsened pain conditions, wrong usage of medication or inadequate medication. In order to minimize the number of adverse side effects patients may also choose to take a lower dosage in order to manage both their level of pain and the adverse side effects. However, giving patients the possibility to address their pain on a regular basis might help to uncover such conflicting circumstances. Maxwell et al. [32] focused on prevalence of pain among nursing home residents and reported one-fifth of the residents with daily pain did not receive analgesics. Also residents aged 75+ years with cognitive impairment or the requirement of an interpreter were significantly less likely to receive an opioid alone or in combination with a non-opioid. This highlights the important fact that changes in cognitive state may hinder the detection of pain and alternative ways to assess the level of pain are needed for patients with cognitive impairment.

In relation to the cognitive state of the participants our results are partly contrary compared to existing literature. Bauer et al. [31] reported higher pain prevalence and less pain related medication for MCI. A pilot study by Monroe et al. [33] indicated greater pain intensity while our data showed significantly lower prevalence of pain for care home settings for MCI participants.

### Depression

As for depression our results demonstrated consistent associations with pain and depression as found by a number of previous studies with younger participants [8, 34, 35]. Throughout the data pain and impairments due to pain and depression were highly correlated for very old people. As de Wall [35] showed perceived control plays a crucial role as mediator between pain and the presence of depressive disorders. With growing age perceived control will very likely decline and support will be needed in physical, psychological, medical and social areas of life. Therefore the group of the very old could be considered to be under greater risk of depression and preventive measure should be put in place.

### Strength and limitations

The strength of our study lies in the large number of participants aged 85+ who gave extensive insight in their medical, social and psychological aspects of life. The large sample size allowed us to look closer at the connection between cognitive state and the housing arrangement which is becoming more important due to the growing number of individuals using care services.

However, we cannot exclude bias. We focused on pain at the point of time while the interview was performed but did not distinguish between chronic and acute pain in our assessments. The pain assessment was carried out using a simple question. It can be speculated whether the test was suitable for this MCI participants though all participants were verbally able to respond. Also we assessed the medication taken regularly and on demand but analgesics taken in the morning or analgesics not used despite being prescribed could have changed the evaluation. A question whether the medication has been taken in the morning was not included in the assessment. We also did not look at specific pain sites because the interview was already quite extensive.

In order to group the participants into the forms of housing we used the level of care giving through a medical assessment carried out by authorities as an indication which excluded participants receiving support through family members only.

### Implications for clinicians and research

The results of the study show that pain scores decrease with age yet the reasons for the results have not been uncovered and can only be speculated upon. In order to explain the differences regarding the housing situation further investigation on the assessment of pain in ambulatory care settings is needed. The integration of routine pain assessments in both ambulatory and care home settings could help to overcome the differences in care settings and lower the number of patients suffering from pain despite taking analgesics. It remains unclear to what extend pain assessments are carried out in the ambulatory care setting in Germany. A general evaluation on this topic may help to identify key aspects in this area. In order to support caring relatives a form of special training or an advisory board may help to sensitize on the topic. For patients with MCI pain assessments should be adapted in order to ensure an accurate treatment and successful aging.

## Conclusions

In conclusion, our results give new insight into prevalence of pain and impairment of activities of daily living for the oldest age groups of our society. Even though pain ratings decrease with age pain is still highly prevalent and successful management of pain is indispensable. Especially women and individuals with comorbidities such as depression are more likely to experience pain in very old age. The appropriate utilization of analgesics remains a problematic issue for individuals affected by pain. Also, the form of housing arrangement chosen in late life seems to be associated with the prevalence of pain. Individuals receiving outpatient care experience significantly more pain. These differences point to a gap in pain management especially in outpatient care settings. This could be due to a lack of interaction between care staff and general practitioners or missing standards on pain evaluation. Considering the growing number of individuals aged 85+ the group of the oldest-old shows an increasing demand of care support including management and treatment of pain.

## Abbreviations

AgeCode: German Study on Ageing, Cognition and Dementia in Primary Care Patients
AgeQualiDe: Study on needs, health service use, costs and health-related quality of life in a large sample of oldest-old primary care patients
BMI: Body Mass Index
CI: Confidence interval
MCI: Mild Cognitive Impairment
NCI: No Cognitive Impairment
OR: Odds ration
PRS: Pain Rating Scale
